# Efficacy and Safety of Nanoadministration in the Treatment of Non-Small-Cell Lung Cancer Is Good to Some Extent: A Systematic Review and Meta-Analysis

**DOI:** 10.1155/2022/9017198

**Published:** 2022-03-08

**Authors:** Rui Han, Youhong Guan, Min Li, Aiqun Xu, Dong Wu, Pulin Li, Enze Wang, Peng Sun, Guanghe Fei, Sijing Zhou, Ran Wang

**Affiliations:** ^1^Department of Respiratory and Critical Care Medicine, The First Affiliated Hospital of Anhui Medical University, Hefei 230022, China; ^2^Department of Infectious Disease, Hefei Second People's Hospital, 230022 Hefei, China; ^3^Department of Oncology, The First Affiliated Hospital of Anhui Medical University, Hefei 230022, China; ^4^Department of General Medicine, Hefei Second People's Hospital, Changjiang East Road, 230022 Hefei, China; ^5^Hefei Third Clinical College of Anhui Medical University, Hefei 230022, China; ^6^Hefei Prevention and Treatment Center for Occupational Diseases, Hefei 230022, China

## Abstract

**Purpose:**

The purpose of this study was to evaluate the efficacy and safety of a nanodrug delivery regimen compared with conventional drug administration for the treatment of lung cancer.

**Materials and Methods:**

Studies were retrieved through PubMed, Web of Science, and ScienceDirect. Primary and secondary outcome measures, including overall response rate (ORR), progression-free survival (PFS), overall survival (OS), and adverse events, were extracted from the retrieved literature and systematically evaluated.

**Results:**

Six trials, including 4806 advanced non-small-cell lung cancer patients, were included in this study. Compared with conventional drug administration in the treatment of lung cancer, the nanodrug delivery regimen improved the ORR (risk ratio = 1.43, 95% confidence interval (CI) = 1.25–1.63, *p* ≤ 0.001), prolonged PFS (hazard ratio (HR) = 0.83, 95% CI = 0.76–0.92, *p* ≤ 0.001), and obtained superior OS (HR = 0.91, 95% CI = 0.83–0.99, *p* ≤ 0.001). Regarding safety, the incidence of neutropenia, alopecia, sensory neuropathy, myalgia, and arthralgia was lower in the nanoadministration group, but the risk of thrombocytopenia, anaemia, and nausea was increased.

**Conclusion:**

Nanodrug administration is safe and effective in patients with non-small-cell lung cancer to some extent.

## 1. Introduction

Lung cancer is the second most frequently diagnosed cancer and the leading cause of cancer deaths worldwide in 2020, accounting for approximately one in nine cancers (11.4%) and one in six deaths (18.0%), with an estimated 2.2 million new cancer cases and 1.8 million deaths [1].

According to histological classification, lung cancer can be divided into epithelial tumors, mesenchymal tumors, lymphohistiocytic tumors, tumors of ectopic origin, and metastatic tumors [2]. Generally, lung cancer statistics include small-cell carcinoma and non-small-cell lung carcinoma (NSCLC). About 13% of lung cancers are small-cell carcinoma and 84% are non-small-cell lung carcinoma according to the American Cancer Society [3], and they are highly heterogeneous. NSCLC can be further classified into specific pathologic subtypes (such as adenocarcinoma and squamous-cell carcinoma) [2]. Five-year survival rates for lung cancer patients in most countries range from 10% to 20%, depending on stage and region [1, 4].

Treatment for lung cancer usually involves a combination of surgery, chemotherapy, and/or radiation, depending on the type of malignancy and stage at diagnosis [5]. However, due to the lack of early diagnosis, most lung cancers are only found in late stages of local tumor invasion or distant metastasis and are not suitable for surgical treatment. A comprehensive study from the TYROL registry has shown that the majority of patients with NSCLC are already at an advanced stage at the time of initial diagnosis and have multiple comorbidities [6], which limits treatment options. Therefore, systemic chemotherapy for the majority of lung cancers is currently the main means of treatment for advanced lung cancer aimed at prolonging survival and improving quality of life [7]. In terms of treatment, immunotherapy alone or in combination has been shown to have a survival benefit for lung cancer patients [8]. Programmed cell death protein 1(PD-1) inhibitors are better than chemotherapy in patients with advanced NSCLC [9]. In patients with advanced NSCLC who use PD-1/programmed cell death 1 ligand 1 (PD-L1) inhibitors, PD-L1 expression is a predictive biomarker for ORR [10]. However, all of these treatments have some limitations. As for conventional administration, it generally refers to oral or intravenous injection of anticancer drugs. Conventional chemotherapeutic agents are nonspecific in distribution in the body; they affect cancer cells and adjacent normal cells, thus limiting the dose within tumor cells, resulting in suboptimal treatment due to excessive toxicity [11]. The hydrophobic nature of the majority of the cancer chemotherapeutics makes them poorly water soluble and therefore limits their administration at high doses [5, 12].

Furthermore, in the past two decades, the introduction of nanotechnology has broadened research prospects in the field of nanomedicine [13]. Studies have shown that nanoparticles can overcome biophysical and biochemical obstacles, improve bioavailability, target, and reduce cytotoxicity. Various treatment methods based on nanoparticles have good efficacy and low toxicity in the treatment of lung cancer [14]. Currently, the nanocarriers of lung cancer drug delivery systems mainly include lipid-based (liposomes and solid lipid nanoparticles), polymer-based (polymeric nanoparticles (NPs)–nanocapsules and nanospheres, dendrimers, and polymeric micelles), metal-based, and magnetic NPs [11]. Yong Il Park et al. successfully developed folate receptor beta (FR*β*)-targeted *pondus hydrogenii* (pH)-sensitive liposomes using docetaxel (DTXL) and doxycycline (DOXY) for the effective treatment of non-small-cell lung cancer [15]. Caina Xu et al. found that doxorubicin and cisplatin nanoparticles had better efficacy than doxorubicin (DOX) or cis-platinum (CDDP) monotherapy for metastatic lung cancer [16]. The results of Shuzhen Chen et al. showed that ferroferric oxide (Fe_3_O_4_) magnetic nanoparticles (MNPs)-targeted delivery of small interfering RNA against baculoviral IAP repeat containing 5 (siBIRC5) and oligodeoxynucleotide antisense (AS-ODN) can enhance radiosensitivity, providing an innovative solution for patients with lung adenocarcinoma who are currently clinically resistant to radiotherapy and have low toxicity risk [17]. In the treatment of lung cancer, the application of nanomedicine is mainly divided into nanomedicine and anticancer drugs, nanomedicine and lung cancer immunotherapy, and active targeting of nanomedicines for lung cancer three aspects [18]. Due to the characteristics of the nanodrug delivery system, it can help overcome the disadvantage of poor water solubility of drugs and improve the biological distribution of drugs [18]. Previous studies have shown that compared with conventional drug administration in the treatment of lung cancer, nanodrug administration is more efficient, less toxic, and more convenient to use (e.g., nano-albumin-bound paclitaxel) [19–21]. A study conducted by Mahsa Shahriari et al. showed that nanodrug delivery could improve the clinical outcome of NSCLC patients [22]. However, these studies included a small sample size and some included only older adults; therefore, the accuracy and conclusions of the studies may not be comprehensive enough, requiring confirmation.

In this study, six studies were included to evaluate the efficacy and safety of nanodosing versus conventional dosing for lung cancer.

## 2. Materials and Methods

This systematic review and network meta-analysis was registered under the PROSPERO platform (#CRD42021268340). We completed this meta-analysis following the guidelines for systematic reviews and meta-analysis, the PRSIMA 2009 checklist, and the preferred reporting items in the Cochrane Handbook. The questions studied in this study follow the principles of population, intervention, comparison, results, and study design. The population included patients with non-small-cell carcinoma and lung cancer. The intervention used is nanoadministration, and the control is conventional administration. The outcome measures included overall response rate (ORR), progression-free survival (PFS), and overall survival (OS). The type of experimental design included in this study is a randomized controlled trial (RCT).

### 2.1. Search Strategy

Electronic databases (PubMed, ScienceDirect, and Web of Science) were systematically searched to extract eligible literature from database inception to November 2021. The literature search process was established using the following keywords and related medical subject headings terms: ‘lung neoplasms' and ‘nanotechnology' or ‘nanomedicine' or ‘nano-delivery systems' or ‘nanoparticles' or ‘NPs' or ‘nano-DDS'. The searches were rerun prior to the final analysis, in English, with publication limited until November 2021.

### 2.2. Selection Criteria

The following were the inclusion criteria: 1. the disease was confirmed to be NSCLC based on histology or cytology; 2. all included studies were clinical trials or RCTs; 3. the results included ORR, PFS, OS, estimated risk ratio (RR), or hazard ratio (HR), 95% confidence interval (CI); 4. the General Terminology Criteria for Adverse Events (CTCAE) version 4.0 (Common Terminology Criteria for Adverse Events (CTCAE) (nih.gov https://evs.nci.nih.gov/ftp1/CTCAE/CTCAE_4.03/CTCAE_4.03_2010-06-14_QuickReference_8.5x11.pdf)) was used to record and classify adverse events; and 5. only documents that can be found in full text can be included.

The exclusion criteria were as follows: 1. inability to extract data; 2. repetition of content; 3. the studies were systematic reviews, meta-analyses, case reports, or retrospective studies; and 4. complications with other neoplastic diseases.

### 2.3. Quality Assessment of the Studies

**Figure 1:**
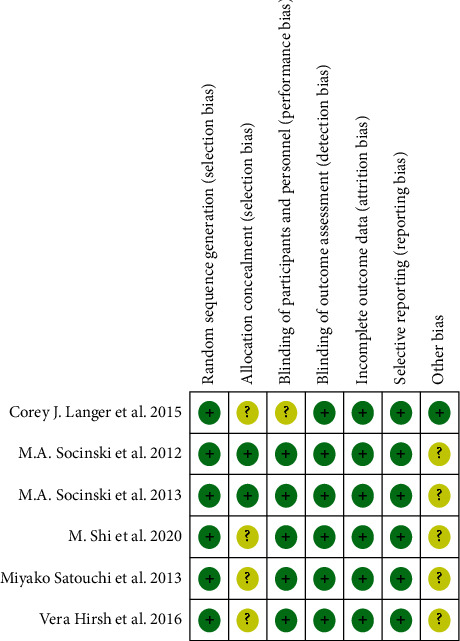
Risk of bias graph: review of authors' judgements about each risk of bias item presented as percentages across all included studies. Note: each colour represents a different level of bias: red for high risk, green for low risk, and yellow for unclear risk of bias.

The risk of bias in the included studies was assessed using the Cochrane Intervention Systems Review Manual (cochrane.pdf [iums.ac.ir]). There are 6 evaluation criteria: (1) random sequence generation; (2) allocation concealment; (3) blindness of participants and researchers; (4) blindness of achievement assessment; (5) incomplete outcome data; and (6) selective reporting. Each term was identified as low, unclear, or high risk of bias (Figure 1).

### 2.4. Data Extraction

Three researchers participated in the data extraction. One researcher extracted baseline data from the literature that met the inclusion criteria, including study publication date, first author, number of patients, age, clinical stage of lung cancer, treatment regimens, and outcome measures (RRs and 95% CI for ORR, HRs for PFS and OS, and number of adverse events of grade 3 or greater). Another researcher examined this issue. Any dispute was resolved after discussion with a third researcher. If there were no data in the paper, the researchers emailed the study authors, asking for raw data or relevant information. The extracted data were recorded in an Excel spreadsheet and processed using Stata 16.0, developed by the U.S. Computer Resource Center.

### 2.5. Statistical Analysis

Statistical analysis was performed using Stata version 16.0. The dichotomous variables, including ORR and adverse event incidence, were evaluated with a 95% CI, RR, or odds ratio (OR). Time event variables, including OS and PFS, were evaluated against the HR. The hypothetical test results for each variable are listed on a forest map. A sensitivity analysis was conducted for outcome indicators with significant heterogeneity, and one inclusion study was excluded at a time to determine the source of heterogeneity. Heterogeneity was assessed using the *χ*^2^ test. If significant heterogeneity was detected (*I*^2^ > 50% or *p* < 0.1), a random-effects model was used; otherwise, a fixed-effects model was used. Statistically significant differences were represented by *p* values < 0.05, and 95% CIs that did not overlap. Funnel plots were used to assess publication bias.

## 3. Results

### 3.1. Study Selection

**Figure 2:**
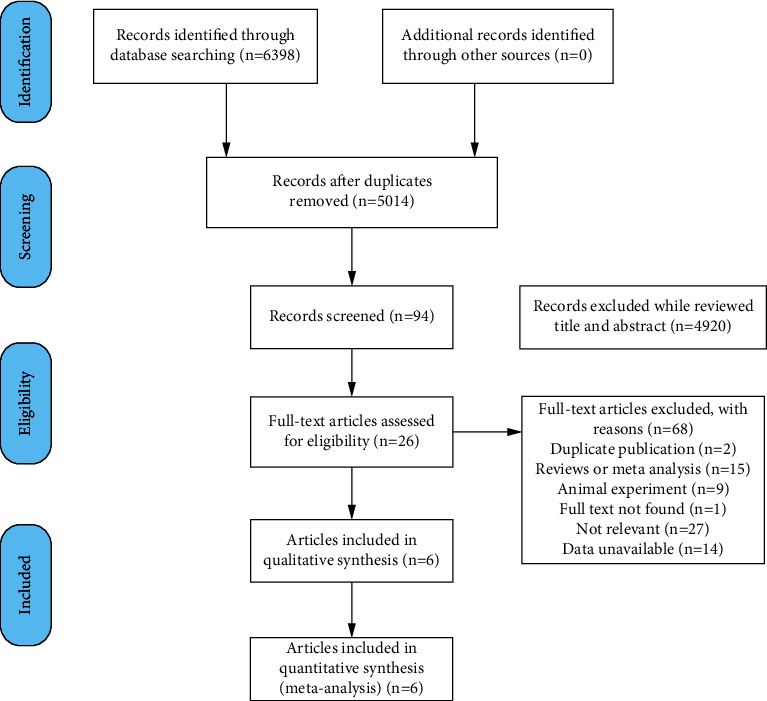
Flowchart of literature screening.

A flow chart outlining the selection process for inclusion in the meta-analysis is shown in Figure 2.

### 3.2. Characteristics of Included Studies

Through the procedure outlined in Figure 2, we identified six articles that met the inclusion criteria. The characteristics of the studies are listed in Table 1.

### 3.3. Outcome Measures

#### 3.3.1. Objective Response Rate

**Figure 3:**
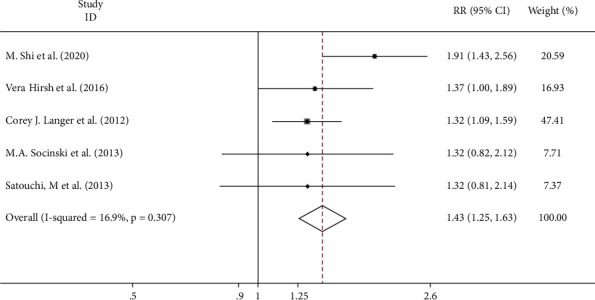
Meta-analysis of the objective response rate (ORR) correlation between nanodrug administration and conventional drug administration.

Five studies reported ORR. The combined estimate showed that there was no significant heterogeneity between the trials (*I*^2^ = 16.9%, *p*=0.307). Therefore, the fixed-effect model was adopted. The fixed-effect model revealed that compared with conventional drug administration, nanoadministration in the treatment of lung cancer improved the ORR (RR = 1.43, 95% CI = 1.25–1.63, *p* ≤ 0.001) (Figure 3).

#### 3.3.2. Progression-Free Survival

**Figure 4:**
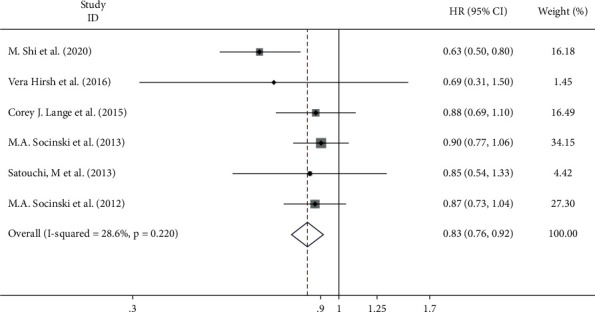
Meta-analysis of the progression-free survival (PFS) correlation between nanodrug administration and conventional drug administration.

All six studies reported PFS. The pooled estimate showed that there was no significant heterogeneity between trials (*I*^2^ = 28.6%, *p*=0.220). No obvious heterogeneity was observed among the experiments. Therefore, the fixed-effect model was used for the meta-analysis. The results of total PFS after combination therapy showed significant differences between the experimental and control groups (HR = 0.83, 95% CI = 0.76–0.92, *p* ≤ 0.001) (Figure 4), which means that compared with conventional drug administration, nanoadministration prolonged PFS.

#### 3.3.3. Overall Survival

**Figure 5:**
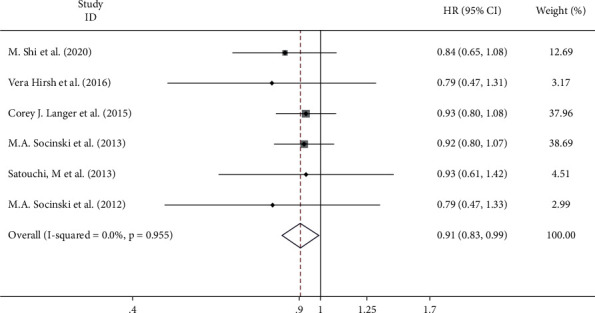
Meta-analysis of the overall survival (OS) correlation between nanodrug administration and conventional drug administration.

OS was reported in all six studies. Since there was no significant heterogeneity among the studies, the fixed-effect model was used for the calculation (*I*^2^ = 0, *p*=0.955). The results showed that there were significant differences in the effects of nanodrug delivery on OS between the two groups (HR = 0.91, 95% CI = 0.83–0.99, *p* ≤ 0.001) (Figure 5). The analysis results showed that compared with conventional administration, nanoadministration resulted in superior OS.

#### 3.3.4. Adverse Events

**Figure 6:**
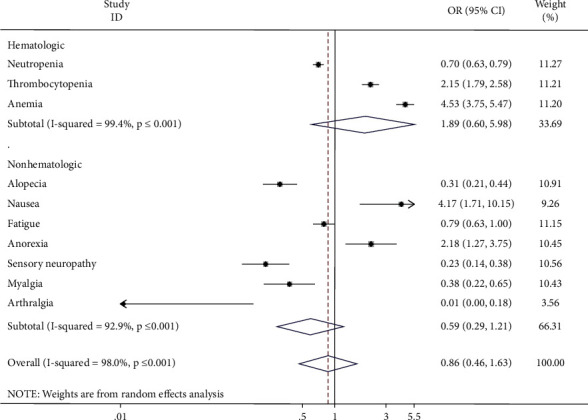
Combined odds ratio (OR) and 95% confidence interval of adverse events included in six trials.

As shown in Figure 6, patients with lung cancer treated with nanomaterials had fewer adverse reactions, such as neutropenia, alopecia, sensory neuropathy, myalgia, and arthralgia (neutropenia: OR = 0.70, 95% CI = 0.63–0.79, *p* ≤ 0.001; alopecia: OR = 0.31, 95% CI = 0.21–0.44, *p* ≤ 0.001; sensory neuropathy: OR = 0.23, 95% CI = 0.14–0.38, *p* ≤ 0.001; myalgia: OR = 0.22, 95% CI = 0.38–0.65, *p*=0.001; and arthralgia: OR = 0.01, 95% CI = 0.00–0.18, *p*=0.001), compared with conventional administration. However, nanoadministered drugs also led to higher rates of thrombocytopenia, anaemia, and nausea (thrombocytopenia: OR = 2.15, 95% CI = 1.79–2.58, *p* ≤ 0.001; anaemia: OR = 4.53, 95% CI = 3.75–5.47, *p* ≤ 0.001; and nausea: OR = 4.17, 95% CI = 1.71–10.15, *p*=0.002). Another adverse effect, fatigue (OR = 0.79, 95% CI = 0.63–1.00, *p*=0.051), did not differ significantly between the two treatment groups.

#### 3.3.5. Publication Bias

**Figure 7:**
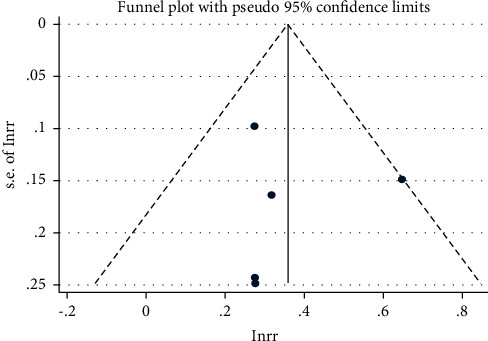
Funnel plot of the risk ratio (RR) of the overall response rate (ORR).

**Figure 8:**
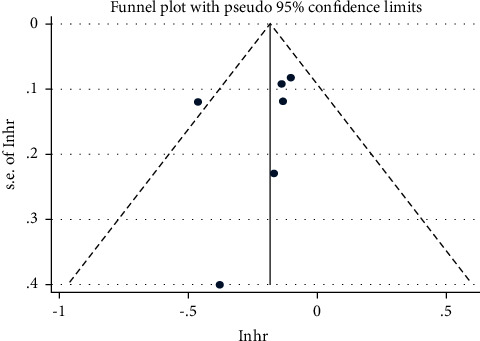
Funnel plot of the hazard ratio (HR) of progression-free survival (PFS).

**Figure 9:**
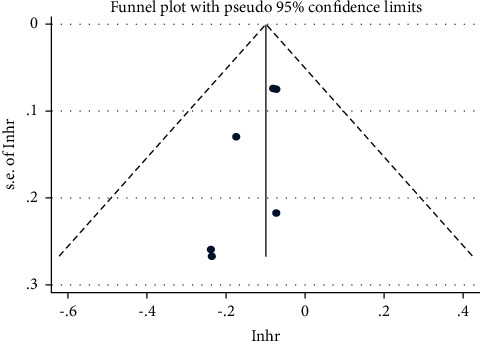
Funnel plot of the hazard ratio (HR) of overall survival (OS).

The publication bias of the primary outcomes (ORR, PFS, and OS) was assessed and represented using a funnel plot. All the graphs were approximately symmetric, indicating that publication bias was well controlled and the reliability was satisfactory (Figures 7–9).

## 4. Discussion

According to the global cancer statistics in 2020, the number of lung cancer deaths accounts for 18.0% of the total number of cancer deaths, which is the main cause of cancer deaths [1]. Studies have shown that smoking, chronic lung disease, air pollution, occupational exposure factors, people with NAT2 nonrapid (slow intermediate) phenotype, and low education levels have a significantly increased risk of lung cancer [29, 30]. Once squamous non-small-cell lung cancer relapses, the treatment options are limited, and elderly patients often do not get adequate treatment because of toxicity problems. However, nab-paclitaxel monotherapy given once a week is effective and safe for this population [31]. The abovementioned statistics show that current treatment for lung cancer still needs to be improved. For most lung cancers, systemic chemotherapy is the mainstay of treatment for advanced lung cancer, with the goal of prolonging survival and improving quality of life. Paclitaxel (PTX) and the water-soluble drug doxorubicin (Dox) are commonly used chemotherapy drugs for lung cancer, but their poor water solubility, low bioavailability, and toxicity are high; therefore, the side effects of serious accessories must be used. For example, lipid-based nanoparticles (in the form of a bilayer structure (liposomes)) or solid core lipid nanoparticles can be used as drug carriers [32]. Currently, PTX nanobiotics are in clinical use as the first approved nanobiotics for the treatment of NSCLC. They improve efficacy and lower blood pressure. In addition, studies have shown that patients can receive higher doses of PTX after encapsulation in nanoscale polymer micelles without increased toxicity [33].

In this study, we included six high-quality clinical trials involving 4806 NSCLC patients aged 18–84 years. Based on these trials, we systematically analysed the differences in ORR, PFS, OS, and adverse events between the nanoadministration and conventional administration groups. According to a 2020 draft guideline for cancer drug approval in clinical trials (FDA Draft Guidance for Clinical Research of Cannabis and Cannabis-Related Products–Global Cannabis Compliance Blog (bakermckenzie.com https://globalcannabiscompliance.bakermckenzie.com/2020/07/26/fda-draft-guidance-for-clinical-research-of-cannabis-and-cannabis-related-products/)), the following three efficacy endpoints are recommended for evaluation: ORR, PFS, and OS. Therefore, we used ORR, PFS, and OS as the evaluation indexes of curative effect in this meta-analysis. The results showed that the nanometer administration group improved the ORR and prolonged PFS and obtained superior OS. In the systematic review of ORR, as shown in the forest map, there are two articles in the original study showing that nanodrug administration has a positive effect on NSCLC and can improve the total response rate of patients. Three of them reported no significant difference between nanodrug administration and ordinary drug administration. However, according to our meta-analysis results, statistical analysis of the results of five studies showed that nanodrug administration was of positive significance in improving the total response rate of NSCLC patients. Similar results were obtained in the PFS analysis. Five articles in the original literature considered that there was no difference in improving PFS between nanodrug administration and ordinary drug administration, but combining the data of six studies, we concluded that nanodrug administration was of positive significance in improving the progression-free survival of patients. Similarly, in the OS analysis, we found that the results of six original studies showed that nanodrug administration could not prolong the OS time of patients with NSCLC. However, combined with all the experimental data and systematic analysis, we can conclude that nanodrug administration can prolong the survival time of patients compared with general drug administration.

The results of M. Shi et al. (2020) [23] showed that compared with Sb-P/C, Pm-P/C significantly improved ORR and PFS in patients with advanced NSCLC, but there was no significant difference in OS. Subgroup analysis showed that Sb-P/C has PFS and OS advantages over Pm-P/C in patients with PS (performance status) 0. However, the results of the subgroup analysis should be carefully interpreted because the sample size of PS 0 patients was very small, and the analysis was not predesigned in this phase III study. In our analysis, the results of M. Shi et al. (2020) showed significant differences in ORR and PFS, which were different from the results of the other five RCT analyses, which may be related to the fact that the research object was Chinese and the experimental group intervention was Pm-P/C. As a nanodrug, polymeric micellar paclitaxel (pm-Pac) could passively target tumors by enhancing permeability and retention effects, significantly reducing the retention of paclitaxel in the blood, thereby enhancing drug uptake and accumulation in tumor tissues [23, 34, 35]. Vera Hirsh et al. (2016) [24] concluded that nab-paclitaxel plus carboplatin (nab-P/C) demonstrated improved efficacy and manageable tolerability in patients with advanced NSCLC and diabetes. Nab-P/C treatment resulted in a significantly higher ORR and longer PFS. After adjusting the baseline characteristics of histology, region, stage, race, and age, the difference in the treatment of PFS was still significant. OS has been improved for more than 6 months, but it has not reached statistical significance. In patients without diabetes, compared with Sb-P/C, nab-P/C treatment can also significantly improve ORR and prolong survival, but not significantly. It is not clear whether the albumin formula of albumin-paclitaxel plays a role in the difference in prognosis between diabetic and nondiabetic patients [24]. Among the subjects studied by Vera Hirsh et al. (2016), lung cancer combined with diabetes accounted for half. Although the correlation between the two is still uncertain, studies have proved that patients with cancer and diabetes have a worse prognosis compared with patients with cancer but without diabetes [36–38]. Corey J. Langer et al. (2015) [25] described the outcome that nab-P/C proved beneficial and tolerable in patients with advanced NSCLC and mild and moderate renal impairment. Renal function decreases with age [39], and this problem is particularly relevant to patients with lung cancer. The median age of the patients included in this study is between 57 and 71 years old. Among these subgroups classified according to the degree of renal injury, there was no significant difference in overall survival and progression-free survival between the nab-P/C group and the Sb-P/C group [25]. M. A. Socinski et al. (2013) [26] drew the conclusion that first-line nab-P/C demonstrated a favorable risk-benefit profile in patients with NSCLC regardless of histology. In this study, treatment continued until the disease progressed. Although the trend of improvement in survival rate was not significant, the tolerance of continuous use of Sb-P/C was much lower than that of nab-P/C. The study conducted by Miyako Satouchi et al. (2013) [27] found the ORR, PFS, and OS of the nab-P/C group were better than those of the Sb-P/C group. The patient's baseline and histological features were well balanced between the two arms. However, the sample size of the research was too small, and the results obtained were not comprehensive. M. A. Socinski et al. (2012) [28] published a study that showed nab-P/C was well tolerated as a first-line treatment in elderly patients with non-small-cell lung cancer, improving ORR, PFS, and OS compared with Sb-P/C. The experiment was divided into elderly (≥70 years old) and younger patients. Older patients were underrepresented in clinical trials, and new treatment options for them were limited due to the expected toxic effects. The nanodrugs used in the later five experiments are all nab-paclitaxel. The subjects of M. Shi et al. (2020) and Miyako Satouchi et al. (2013) were all Asians, while the remaining four experimental studies included Europeans, which may make the differences. According to Ezequiel Bernabeu et al. (2017), patients treated with nab-paclitaxel do not need allergy prodrugs or prolonged transfusions, making administration easier and safer. This is not a small problem because patients have a shorter hospital stay and a significantly lower risk of allergic reactions [40].

Individual experiments may draw contradictory conclusions, but through our comprehensive analysis, we can provide a more accurate estimation of the size of the effect, resolve the conflicts between experiments, and yield conclusive results when individual studies are inconclusive [41]. This may be related to the protection of colloidal systems, such as liposomes and nanoparticles, against premature degradation or (chemical) inactivation in the circulation of anticancer drugs, to have a better effect on patients [42]. In fact, nanomedicine as a delivery vehicle for therapeutic molecules can reduce systemic toxicity and improve pharmacokinetics, which has attracted widespread attention in the treatment of many types of cancer [43]. Nanomedicine as a delivery vehicle for therapeutic molecules has several advantages, some of which are as follows: by extending the circulation time of therapeutic molecules and overcoming their limited water solubility to improve pharmacokinetics, while protecting them from premature inactivation or biodegradation [40]; relying on two targeting mechanisms, active and passive, to deliver higher doses of therapeutic molecules near the tumour [44]; and nanoparticles modified by active biomolecules, such as nucleic acids, peptides, sugars, and antibodies, can actively bind to cancer cells and minimise damage to noncancerous cells [45]. Through our study, we determined the positive significance of nanodrug administration in NSCLC patients. This suggests that nanodrug delivery may be an effective method of drug administration, and further experiments are needed to confirm our conclusion. In previous clinical trials, phase II clinical trials have shown that the effective rate of paclitaxel nanoadministration was higher than that of a solvent-based paclitaxel. In addition, phase III clinical trials have shown that the effective rate of paclitaxel nanoadministration plus carboplatin as first-line chemotherapy is significantly higher than that of conventional paclitaxel plus carboplatin [46].

As for treatment-related toxicity, the results of the meta-analysis showed that nanoadministration was more toxic to haematologic diseases and less toxic to nonhaematologic diseases. In terms of haematologic diseases, compared with conventional administration, the incidence of neutropenia induced by nanoadministration was lower, while the incidence of thrombocytopenia and anaemia increased. Considering nonhaematological toxicity, the nanoadministration group caused less alopecia, sensory neuropathy, myalgia, and arthralgia, but increased the risk of nausea. There are many treatment options for advanced NSCLC [47], but maintaining patient quality of life and PS remains an important factor [48]. Neuropathy, myalgia, and arthralgia can reduce PS so much that they can no longer meet their needs of their daily lives. Similar to previous studies, nab-P/C showed lower neurotoxicity and greater survival benefit compared with Sb-P/C [49]. Furthermore, from the data, we found that there were no known reports of deaths associated with nanotherapy. Therefore, we can infer that nanoadministration can improve tolerance, which may allow higher dose transmission and help improve the survival of patients with NSCLC. This shows the good safety and efficacy of the nanoadministration group, which is in line with the previous conclusion of Zhong et al. [18].

Nonetheless, this study also has some limitations. Although the sample size included in the experiment is large, the mode of nanodrug delivery was mainly nano-albumin-binding paclitaxel, and the lack of diversity of drugs will cause potential bias risk. Moreover, only six studies were included in this study, a relatively small number; therefore, more research and a larger sample size are needed to enhance the accuracy of the conclusions. In addition, one of the six trials lacked ORR results in the meta-analysis of outcome indicators. Therefore, we need a richer variety of drugs and more comprehensive experimental data to further verify our results.

## 5. Conclusions

This meta-analysis shows that the preferred mode of administration in patients with NSCLC is nanoadministration, which can improve the ORR, prolong PFS, and obtain superior OS. Nanodrug administration is safe and effective in patients with NSCLC to some extent.

## Figures and Tables

**Table 1:** Characteristics of the included studies.

Author	M. Shi et al. [23]		Vera Hirsh et al. [24]				Corey J. Lange et al. [25]						M. A. Socinski et al. [26]				Satouchi, M et al. [27]		M. A. Socinsk et al. [28]			
Year	2020		2016				2015						2013				2013		2012			
Country	China		Australia, Canada, Japan, Russia, Ukraine, the United States				Australia, North America, Japan, Russia/Ukraine						Australia, Canada, Japan, Russia, Ukraine, USA				Japan		Australia, Canada, Japan, Russia, Ukraine, USA			
Disease	NSCLC		NSCLC with diabetes		NSCLC (CrCl < 50 mL/min)		NSCLC (CrCl 50–80 mL/min)		NSCLC (CrCl 50–80 mL/min)		NSCLC (CrCl >80 mL/min)		NSCLC(SCC)		NSCLC (NSCC)		NSCLC		NSCLC			
Disease stage	IIIB/IV		IIIB/IV		IIIB/IV		IIIB/IV		IIIB/IV		IIIB/IV		III/IV		III/IV		IIIB/IV		IIIB/IV			
Age, median (range), years	60 (25–70)	60 (35–70)	70 (50–80)	71 (51–80)	71 (51–80)	60 (24–84)	70 (50–80)	71 (51–80)	62 (34–81)	64 (40–84)	57 (28–78)	57 (24–77)	NR (28–81)	NR (34–84)	NR (29–79)	NR (24–82)	65 (37–79)	64 (36–77)	58 (28–69)	59 (24–69)	72 (70–81)	72 (70–84)
Treatment	Pm-P/C	Sb-P/C	Nab-P/C	Sb-P/C	Sb-P/C	Sb-P/C	Nab-P/C	Sb-P/C	Nab-P/C	Sb-P/C	Nab-P/C	Sb-P/C	Nab-P/C	Sb-P/C	Nab-P/C	Sb-P/C	Nab-P/C	Sb-P/C	Nab-P/C	Sb-P/C	Nab-P/C	Sb-P/C
Number of patients	300	148	26	27	27	501	26	27	198	206	289	291	229	221	292	310	74	75	447	449	74	82
Endpoint	ORR, PFS, OS		ORR, PFS, OS		ORR, PFS, OS		ORR, PFS, OS		ORR, PFS, OS		ORR, PFS, OS		ORR, PFS, OS		ORR, PFS, OS		ORR, PFS, OS		PFS, OS		PFS, OS	
ORR	50%	26%	31%	27%	31%	25%	31%	19%	35%	27%	32%	25%	41%	24%	26%	25%	35%	27%	32%	25%	34%	24%
RR	1.91		1.66		1.27		1.66		1.32		1.29		1.68		1.03		1.32		NR		NR	
95% CI	[1.43, 2.56]		[0.624, 4.423]		[1.039, 1.553]		[0.624, 4.423]		[0.986, 1.777]		[0.990, 1.672]		[1.271, 2.221]		[0.788, 1.358]		[0.810, 2.143]		NR		NR	
PFS (months)	6.4	5.3	6	4.9	6	5.8	6	4.7	7.8	5.7	5.6	6.4	5.6	6.9	6.9	6.5	6.9	5.6	6	5.8	8	6.8
HR	0.63		0.607		0.95		0.607		0.78		1.012		0.865		0.933		0.845		0.903		0.687	
95% CI	[0.50, 0.80]		[0.269, 1.370]		[0.801, 1.116]		[0.269, 1.370]		[0.595, 1.023]		[0.814, 1.257]		[0.680, 1.101]		[0.750, 1.159]		[0.539, 1.325]		[0.759, 1.074]		[0.420, 1.123]	
OS (months)	18	16.4	9.7	11.1	11.6	11.2	9.7	9.3	13.8	12.4	11.9	11.5	10.7	9.5	13.1	13	16.7	15.9	11.4	11.3	19.9	10.4
HR	0.84		0.824		0.95		0.824		0.853		0.995		0.89		0.95		0.93		0.999		0.583	
95% CI	[0.65, 1.08]		[0.418, 1.624]		[0.817, 1.101]		[0.418, 1.624]		[0.672, 1.082]		[0.819,1.209]		[0.719, 1.101]		[0.779, 1.158]		[0.608, 1.425]		[0.855, 1.167]		[0.388, 0.875]	

ORR, objective response rate; RR, risk ratio; PFS, progression-free survival; HR, hazard ratio; OS, overall survival; 95% CI, 95% confidence interval; NSCLC, non-small-cell lung cancer; SCC, squamous cell; NSCC, nonsquamous cell; Pm-P/C, polymeric micellar paclitaxel plus cisplatin; Sb-P/C, solvent-based paclitaxel plus cisplatin; Nab-P/C, nab-paclitaxel plus carboplatin; NR, not reported; CrCl, creatinine clearance.

## Data Availability

All the data generated or analysed during this study are included in this published article.

## Supplementary Materials

Supplementary Materials The PRSIMA 2009 checklist is used to improve clarity and transparency.
